# RETRACTION: Adiponectin Protects Skeletal Muscle From Ischaemia–Reperfusion Injury in Mice through miR‐21/PI3K/Akt Signalling Pathway

**DOI:** 10.1111/iwj.70562

**Published:** 2025-04-18

## Abstract

**RETRACTION:** ZhouM. , ZhangH. , ChenH. , and QiB. , “Adiponectin Protects Skeletal Muscle From Ischaemia–Reperfusion Injury in Mice through miR‐21/PI3K/Akt Signalling Pathway,” International Wound Journal 20, no. 5 (2023): 1647–1661, 10.1111/iwj.14022.36426910 PMC10088838

The above article, published online on 25 November 2022, in Wiley Online Library (http://onlinelibrary.wiley.com/), has been retracted by agreement between the journal Editor in Chief, Professor Keith Harding; and John Wiley & Sons Ltd. Following an investigation by the publisher, all parties have concluded that this article was accepted solely on the basis of a compromised peer review process. The editors have therefore decided to retract the article. The authors did not respond to our notice regarding the retraction.

**RETRACTION:** M. Zhou , H. Zhang , H. Chen , and B. Qi , “Adiponectin Protects Skeletal Muscle From Ischaemia–Reperfusion Injury in Mice through miR‐21/PI3K/Akt Signalling Pathway,” International Wound Journal 20, no. 5 (2023): 1647–1661, 10.1111/iwj.14022.36426910 PMC10088838

The above article, published online on 25 November 2022, in Wiley Online Library (http://onlinelibrary.wiley.com/), has been retracted by agreement between the journal Editor in Chief, Professor Keith Harding; and John Wiley & Sons Ltd. Following an investigation by the publisher, all parties have concluded that this article was accepted solely on the basis of a compromised peer review process. The editors have therefore decided to retract the article. The authors did not respond to our notice regarding the retraction.

